# Dead space ventilation-related indices: bedside tools to evaluate the ventilation and perfusion relationship in patients with acute respiratory distress syndrome

**DOI:** 10.1186/s13054-023-04338-4

**Published:** 2023-02-03

**Authors:** Mingjia Zheng

**Affiliations:** Department of Respiratory and Critical Care Medicine, Huzhou Central Hospital, Affiliated Central Hospital Huzhou University, No. 1558, Sanhuan North Road, Wuxing, Huzhou, Zhejiang People’s Republic of China

**Keywords:** Acute respiratory distress syndrome, Dead space, Physiological dead space fraction, Ventilatory ratio, End-tidal-to-arterial PCO_2_ ratio, Ventilation and perfusion mismatch

## Abstract

Cumulative evidence has demonstrated that the ventilatory ratio closely correlates with mortality in acute respiratory distress syndrome (ARDS), and a primary feature in coronavirus disease 2019 (COVID-19)-ARDS is increased dead space that has been reported recently. Thus, new attention has been given to this group of dead space ventilation-related indices, such as physiological dead space fraction, ventilatory ratio, and end-tidal-to-arterial PCO_2_ ratio, which, albeit distinctive, are all global indices with which to assess the relationship between ventilation and perfusion. These parameters have already been applied to positive end expiratory pressure titration, prediction of responses to the prone position and the field of extracorporeal life support for patients suffering from ARDS. Dead space ventilation-related indices remain hampered by several deflects; notwithstanding, for this catastrophic syndrome, they may facilitate better stratifications and identifications of subphenotypes, thereby providing therapy tailored to individual needs.

## Introduction

A hallmark of classical ARDS is an increased shunt caused by alveolar collapse and/or alveolar flooding from a physiological viewpoint [1]. Over the past two decades, there has been increasing interest in dead space since the publication by Nuckton et al. in the early twenty-first century [2]. Indeed, the Berlin definition was based on ${\text{PaO}}_{2} /{\text{FiO}}_{2}$(i.e., arterial partial pressure of ${\text{O}}_{2}$ to fraction of inspired ${\text{O}}_{2}$) to classify patients into three categories, but its predictive power for mortality was far from perfect [3, 4]. Given that increased dead space was not uncommon in patients with ARDS and its association with reduced survival [2], ${\dot{\text{V}}\text{E}}_{{{\text{CORR}}}}$ (i.e., the corrected minute ventilation) (Tables 1 and 2) serving as a substitute for dead space, was used to define the severe ARDS subgroup in the draft Berlin definition; nevertheless, this failed. Thus, the final Berlin definition did not incorporate ${\dot{\text{V}}\text{E}}_{{{\text{CORR}}}}$ [3]. Moreover, dead space has been suggested to be predominant in COVID-19-ARDS [5]. Finally, a growing number of intuitive dead space ventilation-related indices with prognostic value have emerged [6, 7]. Therefore, attention has been redirected to these parameters that reflect ventilation and perfusion mismatch.

**Table 1:** Glossary of gas variables and notations

Variables	Definitions	Possible compartment *X*	Example of gas *Y*	Possible subcompartment *Z*
PXY	Partial pressure of gas *Y* in a compartment *X*	${\overline{\text{E}}}$ = mixed expired	CO_2_	
		ET = end-tidal	O_2_	
		A = mean alveolar		
		a = arterial		
VX_Z_	Volume in a compartment *X* and a subcompartment *Z*	T = tidal		phys = physiological
		D = dead space		aw = airway
				anat = anatomic
				inst = instrumental
				alv = alveolar

**Table 2:** Summary of dead space ventilation-related indices

Authors	Index	Advantages	Disadvantages
Bohr [8]	${\text{VD}}_{{{\text{Bohr}}}} /{\text{VT}} = \frac{{{\text{PACO}}_{2} - {\text{P}}\overline{{\text{E}}} {\text{CO}}_{2} }}{{{\text{PACO}}_{2} }}$	Direct and physiologically intuitive to calculate	Relies on Vcap; does not reflect regional ${\dot{\text{V}}\text{A}}/{\dot{\text{Q}}}$ mismatch
Enghoff [9]	${\text{VD}}_{{{\text{B}} - {\text{E}}}} /{\text{VT}} = \frac{{{\text{PACO}}_{2} - {\text{P}}\overline{{\text{E}}} {\text{CO}}_{2} }}{{{\text{PACO}}_{2} }}$	Direct and physiologically intuitive to calculate; a global index to assess ${\dot{\text{V}}\text{A}}/{\dot{\text{Q}}}$ mismatch	Relies on Vcap; does not reflect regional ${\dot{\text{V}}\text{A}}/{\dot{\text{Q}}}$ mismatch
Siddiki et al. [10] and Beitler et al. [11]	$\frac{{{\text{VD}}}}{{{\text{VT}}}} = 1 - \frac{{\left( {{\dot{\text{V}}\text{CO}}_{2} \times 0.863} \right)}}{{\left( {{\text{RR}} \times {\text{VT}} \times {\text{PaCO}}_{2} } \right)}}$ ${\dot{\text{V}}\text{CO}}_{2} = \frac{{{\text{REE}}}}{{\left( {\frac{5.616}{{{\text{RQ}}}} + 1.584} \right)}}$ Uses a rearranged alveolar ventilation equation in combination with indirect measurements of ${\dot{\text{V}}\text{CO}}_{2}$, and modified Harris–Benedict equation, or unadjusted Harris–Benedict equation, or Penn state equation is used to estimate REE	Does not rely on Vcap; considers the influence of ${\dot{\text{V}}\text{CO}}_{2}$, especially suitable for patients treated with ECLS; a global index to assess ${\dot{\text{V}}\text{A}}/{\dot{\text{Q}}}$ mismatch	Predictive power is limited especially due to estimate ${\dot{\text{V}}\text{CO}}_{2}$ indirectly; changes in ${\dot{\text{V}}\text{CO}}_{2}$ due to ECLS procedures should be consider when make comparisons of VR among individuals; does not reflect regional ${\dot{\text{V}}\text{A}}/{\dot{\text{Q}}}$ mismatch
Beitler et al. [11]	$$\begin{aligned} \frac{{{\text{VD}}}}{{{\text{VT}}}} = & 0.1726 + 0.0059 \times {\text{RR}} + 0.0054 \times {\text{PEEP}} + 0.0293 \\ & \times \;{\text{LIS}} + 0.0036 \times {\text{PaCO}}_{2} \times {\dot{\text{V}}\text{E}} + 0.000057 \\ & \times \;{\text{PaCO}}_{2} \times {\text{age}} \\ \end{aligned}$$	Does not rely on Vcap; a global index to assess ${\dot{\text{V}}\text{A}}/{\dot{\text{Q}}}$ mismatch	Predictive power is limited especially due to use physiological variables and a predictive equation; does not reflect regional ${\dot{\text{V}}\text{A}}/{\dot{\text{Q}}}$ mismatch
Sinha et al. [6]	${\text{VR}} = \frac{{{\dot{\text{V}}\text{E}}_{{{\text{measured}}}} \times {\text{PaCO}}_{{2{\text{measured}}}} }}{{{\dot{\text{V}}\text{E}}_{{{\text{predicted}}}} \times {\text{PaCO}}_{{2{\text{predicted}}}} }}$ or ${\text{VR}} = \frac{{{\dot{\text{V}}\text{CO}}_{2} \times 0.863}}{{\left( {1 - \frac{{{\text{VD}}}}{{{\text{VT}}}}} \right) \times \left( {{\text{VE}}_{{{\text{predicted}}}} \times {\text{PaCO}}_{{2{\text{predicted}}}} } \right)}}$ ${\text{VE}}_{{{\text{predicted}}}} = 100{\text{mL}}\cdot{\text{min}}^{ - 1} \times {\text{PBW}}$, ${\text{PaCO}}_{{2{\text{predicted}}}} = 37.5{\text{mmHg}}$	Does not rely on Vcap; considers the influence of ${\dot{\text{V}}\text{CO}}_{2}$, especially suitable for patients treated with ECLS; a global index to assess ${\dot{\text{V}}\text{A}}/{\dot{\text{Q}}}$ mismatch	Changes in ${\dot{\text{V}}\text{CO}}_{2}$ due to ECLS procedures should be consider when make comparisons of VR among individuals; does not reflect regional ${\dot{\text{V}}\text{A}}/{\dot{\text{Q}}}$ mismatch
The ARDS Berlin Definition Task Force [3]	${\dot{\text{V}}\text{E}}_{{{\text{CORR}}}} = {\dot{\text{V}}\text{E}}_{{{\text{measured}}}} \times {\text{PaCO}}_{{2{\text{measured}}}} /40$ or ${\dot{\text{V}}\text{E}}_{{{\text{CORR}}}} = \frac{{{\dot{\text{V}}\text{CO}}_{2} \times 0.863}}{{\left( {1 - \frac{{{\text{VD}}}}{{{\text{VT}}}}} \right) \times 40}}$	Does not rely on Vcap; consider the influence of ${\dot{\text{V}}\text{CO}}_{2}$, especially suitable for patients treated with ECLS; a global index to assess ${\dot{\text{V}}\text{A}}/{\dot{\text{Q}}}$ mismatch	Does not standardize ${\dot{\text{V}}\text{E}}$ for patients’ weight, the accuracy to reflect $\frac{{{\text{VD}}}}{{{\text{VT}}}}$ is limited; changes in ${\dot{\text{V}}\text{CO}}_{2}$ due to ECLS procedures should be considered when make comparisons of VR among individuals; does not reflect regional ${\dot{\text{V}}\text{A}}/{\dot{\text{Q}}}$ mismatch
Gattinoni et al. [7]	$\frac{{{\text{PETCO}}_{2} }}{{{\text{PaCO}}_{2} }}$	Does not rely on Vcap; a global index to assess ${\dot{\text{V}}\text{A}}/{\dot{\text{Q}}}$ mismatch	Assumes ${\text{PETCO}}_{2} = {\text{PACO}}_{2}$; does not include ${\text{VD}}_{{{\text{aw}}}}$; neglects the influences of ${\dot{\text{V}}\text{CO}}_{2}$ and ${\dot{\text{V}}\text{E}}$; does not reflect regional ${\dot{\text{V}}\text{A}}/{\dot{\text{Q}}}$ mismatch

*VD/VT* Dead space fraction, *RR* Respiratory rate, *RQ* Respiratory quotient, $\dot{V}E_{predicted}$ Predicted minute ventilation, *PaCO*_*2predicted*_ Predicted arterial pressure of CO_2_, $\dot{V}E_{measured}$ Measured minute ventilation, *PaCO*_*2measured*_ Measured arterial pressure of CO_2_, *LIS* Murray lung injury score, *PBW* Predicted body weight, other terms of variables see Table 1 and abbreviations

This review covers three dead space ventilation-related indices that have attracted a great deal of attention. After a brief introduction, their current applications are described, and possible physiological rationales are unveiled. Despite several inevitable drawbacks, perhaps in the next decade, these parameters might be used in the subclassifications of ARDS based on severity and help to divide this heterogeneous syndrome into different subphenotypes to better guide personalized treatment management.

## Dead space ventilation-related indices

### Physiological dead space fraction

Dead space or physiological dead space (i.e., ${\text{VD}}_{{{\text{phys}}}}$) is part of the volume that is ventilated but does not participate in gas exchange. ${\text{VD}}_{{{\text{phys}}}}$ can be divided into two components: airway dead space (i.e., ${\text{VD}}_{{{\text{aw}}}}$) and alveolar dead space (i.e., ${\text{VD}}_{{{\text{alv}}}}$). In mechanically ventilated patients, instrumental dead space (i.e., ${\text{VD}}_{{{\text{inst}}}}$) which is the volume related to artificial airway could increase ${\text{VD}}_{{{\text{aw}}}}$. ${\text{VD}}_{{{\text{phys}}}}$ and its subcomponents are commonly expressed as the fraction of tidal volume to allow interpatient comparisons [12, 13]. Christian Bohr proposed a formula in 1891 to calculate dead space. Bohr’s dead space fraction (i.e., ${\text{VD}}_{{{\text{Bohr}}}} /{\text{VT}}$) was calculated in the following manner: ${\text{VD}}_{{{\text{Bohr}}}} /{\text{VT}} = \frac{{{\text{PACO}}_{2} - {\text{P}}\overline{{\text{E}}} {\text{CO}}_{2} }}{{{\text{PACO}}_{2} }}$ (Table 2) [8]. In an ideal lung assuming all units with perfect ${\dot{\text{V}}\text{A}}/{\dot{\text{Q}}}$ matching, ${\text{PACO}}_{2}$ is identical to ${\text{PaCO}}_{2}$ [14]. Thus, in 1938, Enghoff used ${\text{PaCO}}_{2}$ instead of ${\text{PACO}}_{2}$ to modify Bohr’s formula as follows: ${\text{VD}}_{{{\text{B}} - {\text{E}}}} /{\text{VT}} = \frac{{{\text{PACO}}_{2} - {\text{P}}\overline{{\text{E}}} {\text{CO}}_{2} }}{{{\text{PACO}}_{2} }}$ (Table 2) [9]. Enghoff’s modification of Bohr’s dead space fraction (i.e., ${\text{VD}}_{{{\text{B}} - {\text{E}}}} /{\text{VT}}$) represents the physiological dead space fraction (${\text{VD}}_{{{\text{phys}}}} /{\text{VT}}$). However, on the one hand, the blood from ${\dot{\text{Q}}}_{{{\text{VA}}}} /{\dot{\text{Q}}}_{{\text{T}}}$ which consists of true shunt units (i.e., ${\dot{\text{V}}\text{A}}/{\dot{\text{Q}}}$ = 0) and low ${\dot{\text{V}}\text{A}}/{\dot{\text{Q}}}$ units could raise ${\text{PaCO}}_{2}$ [15]; thus, this substitution could increase error of calculating true dead space (i.e., ${\dot{\text{V}}\text{A}}/{\dot{\text{Q}}} = \infty$) and high ${\dot{\text{V}}\text{A}}/{\dot{\text{Q}}}$ units, on the other hand, using $\frac{{{\text{PACO}}_{2} - {\text{P}}\overline{{\text{E}}} {\text{CO}}_{2} }}{{{\text{PACO}}_{2} }}$ considers all forms of ${\dot{\text{V}}\text{A}}/{\dot{\text{Q}}}$ mismatch [16]. Therefore, ${\text{VD}}_{{{\text{phys}}}} /{\text{VT}}$ (i.e., ${\text{VD}}_{{{\text{B}} - {\text{E}}}} /{\text{VT}}$) is a global index with which to assess ${\dot{\text{V}}\text{A}}/{\dot{\text{Q}}}$ mismatch [12]. In 2002, Nuckton et al. first demonstrated that a high ${\text{VD}}_{{{\text{phys}}}} /{\text{VT}}$ was independently associated with an increased risk of death among patients with ARDS [2].

### Ventilatory ratio

It is widely accepted that the presence of true dead space units (i.e., ${\dot{\text{V}}\text{A}}/{\dot{\text{Q}}} = \infty$) and high ${\dot{\text{V}}\text{A}}/{\dot{\text{Q}}}$ units could cause hypercapnia, and an increase in ${\dot{\text{V}}\text{E}}$ could facilitate ${\text{CO}}_{2}$ elimination to maintain an unchanged ${\text{PaCO}}_{2}$, which implies an association between ${\dot{\text{V}}\text{E}}$ and ${\text{PaCO}}_{2}$. Therefore, the ventilatory ratio (VR) was developed to better evaluate ventilatory efficiency. VR is described as ${\text{VR}} = \frac{{{\dot{\text{V}}\text{E}}_{{{\text{measured}}}} \times {\text{PaCO}}_{{2{\text{measured}}}} }}{{{\dot{\text{V}}\text{E}}_{{{\text{predicted}}}} \times {\text{PaCO}}_{{2{\text{predicted}}}} }}$ (Table 2). Likewise, VR reflects a continuous spectrum of ${\dot{\text{V}}\text{A}}/{\dot{\text{Q}}}$ mismatch in the lung [6]. Not surprisingly, authors have also validated that there is an intimate correlation between VR and ${\text{VD}}_{{{\text{phys}}}} /{\text{VT}}$ [17–21], and most studies have concluded that a higher VR is a reliable indicator of mortality [17–22].

### End-tidal-to-arterial PCO_2_ ratio

In the last century, authors calculated the ratio of alveolar dead space to alveolar tidal volume (i.e., $\frac{{{\text{VD}}_{{{\text{alv}}}} }}{{{\text{VT}}_{{{\text{alv}}}} }}$), which equals $\frac{{{\text{P}}\left( {{\text{a}} - {\text{ET}}} \right){\text{CO}}_{2} }}{{{\text{PaCO}}_{2} }}$ and can be restated as $1 - {\text{PETCO}}_{2} /{\text{PaCO}}_{2}$ [23]. Recently, Gattinoni et al. suggested that the end-tidal-to-arterial PCO_2_ ratio $\left( {\frac{{{\text{PETCO}}_{2} }}{{{\text{PaCO}}_{2} }}} \right)$ (Table 2) deriving from $\frac{{{\text{VD}}_{{{\text{alv}}}} }}{{{\text{VT}}_{{{\text{alv}}}} }}$ could be used as a bedside tool to monitor gas exchanges of patients with COVID-19-ARDS. $\frac{{{\text{PETCO}}_{2} }}{{{\text{PaCO}}_{2} }}$ is also a global index with which to assess ${\dot{\text{V}}\text{A}}/{\dot{\text{Q}}}$ mismatch, with a maximum value of 1. When this ratio approaches 1, it reflects an ameliorated gas exchange; conversely, deviation from 1 reflects gas exchange disturbance [7]. Later, authors established that there was a good correlation between $\frac{{{\text{PETCO}}_{2} }}{{{\text{PaCO}}_{2} }}$ and ${\text{VD}}_{{{\text{phys}}}} /{\text{VT}}$; in addition, a reduction in $\frac{{{\text{PETCO}}_{2} }}{{{\text{PaCO}}_{2} }}$ was associated with a higher mortality risk in the non-COVID-19-ARDS and COVID-19-ARDS populations [24, 25].

## Current applications

### PEEP titration

Regarding PEEP titration, intensivists have gradually realized that it is not sufficient to target anatomical recruitment and/or improved oxygenation [26, 27], whereas targeting ${\dot{\text{V}}\text{A}}/{\dot{\text{Q}}}$ matching may represent a promising approach. The gold standard to evaluate ${\dot{\text{V}}\text{A}}/{\dot{\text{Q}}}$ matching is the multiple inert gas elimination technique (MIGET), which is complex [28]. Recently, authors using the automatic lung parameter estimator (ALPE) method found that after increases in PEEP, ${\dot{\text{V}}\text{A}}/{\dot{\text{Q}}}$ matching exhibited heterogeneous responses [29]. Furthermore, based on electrical impedance tomography (EIT), Spinelli et al. reported that measurements of ${\dot{\text{V}}\text{A}}/{\dot{\text{Q}}}$ mismatch allowed the identification of patients with a higher risk of death [30]. Thus, evaluation of ${\dot{\text{V}}\text{A}}/{\dot{\text{Q}}}$ matching likely outperforms the methods that concentrate on anatomic recruitment (i.e., the methods based on respiratory mechanics and morphology) and the method according to oxygenation, thereby playing an important role in determining optimal PEEP.

Almost 50 years ago, Suter et al. first defined the optimal PEEP as that giving rise to the lowest ${\text{VD}}_{{{\text{phys}}}} /{\text{VT}}$ [31]. Furthermore, other studies demonstrated that indices such as the arterial minus end-tidal ${\text{CO}}_{2}$ gradient (i.e., ${\text{P}}\left( {{\text{a}} - {\text{ET}}} \right){\text{CO}}_{2}$) and the ratio of alveolar dead space to alveolar tidal volume (i.e., $\frac{{{\text{VD}}_{{{\text{alv}}}} }}{{{\text{VT}}_{{{\text{alv}}}} }}$) were also helpful in PEEP titration [32–36]. These two indices are analogous to $\frac{{{\text{PETCO}}_{2} }}{{{\text{PaCO}}_{2} }}$; thus, both are associated with dead space, or more specifically ${\dot{\text{V}}\text{A}}/{\dot{\text{Q}}}$ matching. Altogether, employing these dead space ventilation-related parameters may help to titrate the optimal PEEP.

### Prediction of response to the prone position

The PP can exert its impact even without ventilatory support, but the related risks cannot be ignored [37]. Thus, it is crucial to predict which patients with ARDS would benefit from the PP. Traditionally, ${\text{PaO}}_{2} /{\text{FiO}}_{2}$ was conceived of as a better indicator of a positive response to the PP [38]. In the landmark PROSEVA trial, Guerin and colleagues reported that prone positioning for an average of 16 h/d improved oxygenation and reduced the mortality of patients with ARDS by 50% [39]. Nonetheless, the correlation between enhanced survival and improved oxygenation was not significant [40]. Prior to this renowned clinical trial, two study groups reported that positive responses to the PP were better predicted by changes in ${\text{PaCO}}_{2}$ rather than ${\text{PaO}}_{2} /{\text{FiO}}_{2}$ [41, 42]. However, lately, using ${\text{PaO}}_{2} /{\text{FiO}}_{2}$ to determine PP responders was showed to be a reliable indicator of patients who would survive, especially in the literature that focused on selective patients with COVID-19-ARDS [43–45].

Can this contradiction be explained? Enlightened by the findings from Gattinoni et al. [7, 41, 46], the most rational explanation for this contradiction may be as follows: Provided that hemodynamics do not change [47], for a large proportion of COVID-19-ARDS and a lower percentage of classical ARDS patients, an improved ${\text{PaO}}_{2}$ caused by redistributed blood flow indicates success in the PP; in regard to most classical ARDS and remaining COVID-19 ARDS patients, after the PP recruits collapsed or flooded lung units, a fall in ${\text{PaCO}}_{2}$ occurs, albeit in combination with an increased ${\text{PaO}}_{2}$. However, considering the low resistance to diffusion of ${\text{CO}}_{2}$, a change in ${\text{PaCO}}_{2}$ is a more sensitive marker than ${\text{PaO}}_{2}$ [42]. Thus, once a patient exhibits a decreased ${\text{PaCO}}_{2}$, clinicians can identify this patient as a PP responder.

Admittedly, whether ${\text{PaO}}_{2}$ or ${\text{PaCO}}_{2}$ is the best predictor of PP responses is phenotype dependent, and PP responders must correspond to enhanced ${\dot{\text{V}}\text{A}}/{\dot{\text{Q}}}$ homogeneity. Furthermore, using EIT, recent studies confirmed that the PP could improve ${\dot{\text{V}}\text{A}}/{\dot{\text{Q}}}$ matching not only in patients with COVID-19-ARDS but also in non-COVID-19-ARDS patients [48, 49]. Recently, two study groups used VR to determine PP responders [45, 50]. Overall, dead space ventilation-related parameters may be used to predict positive responses during the PP.

### Identifications of candidates for extracorporeal CO_2_ removal (ECCO2R)

In the recent REST trial, in contrast to lung protective ventilation, ECCO2R-facillitated ultraprotective ventilation did not significantly reduce 90-day mortality, but a higher incidence of complications was observed [51]. Thus, weighing the benefits against adverse events and identifying the best candidates for ECCO2R are issues that remain to be addressed [27, 52–54]. Goligher et al. found that an increased ${\text{VD}}_{{{\text{alv}}}} /{\text{VT}}$ could be used to predict a reduced driving pressure and a fall in VT after ECCO2R [55, 56]. The implication of this observation is that for patients with ${\dot{\text{V}}\text{A}}/{\dot{\text{Q}}}$ mismatch resulting from an elevated ${\text{VD}}_{{{\text{alv}}}} /{\text{VT}}$, using ECCO2R to promote ${\text{CO}}_{2}$ removal would be more beneficial.

### Guide to weaning from venovenous extracorporeal membrane oxygenation (vv-ECMO)

The standardized weaning protocol of vv-ECMO for patients with ARDS remains undetermined. Currently, the decision regarding liberation from vv-ECMO is mainly based on oxygenation [57, 58]. However, Al-Fares et al. suggested that VR (i.e., VR > 2.3, sensitivity = 100%, specificity = 81%) could be employed to predict the likelihood of safe liberation from vv-ECMO [59]. More recently, a negative impact of lower baseline $\frac{{{\text{PETCO}}_{2} }}{{{\text{PaCO}}_{2} }}$ on weaning outcome was demonstrated by Lazzari and colleagues. The cutoff value of this parameter was 0.84 (sensitivity = 92%, specificity = 80%) [60]. Hence, the optimal time to safely disconnect a patient from vv-ECMO is when the gas exchange in native lungs is improved, which is signified by dead space ventilation-related indices.

## Limitations

Although dead space ventilation-related indices are promising bedside tools to assess ${\dot{\text{V}}\text{A}}/{\dot{\text{Q}}}$ mismatch, several limitations must be highlighted. In general, these parameters merely indicate overall ${\dot{\text{V}}\text{A}}/{\dot{\text{Q}}}$ mismatch and therefore are not perfect substitutes for more precise techniques, such as EIT [61].

### Physiological dead space fraction

Direct measurements of ${\text{VD}}_{{{\text{phys}}}} /{\text{VT}}$ still rely on volumetric capnography (Vcap), and some technical difficulties limit its widespread use in the clinical setting [62]. Thus, some researchers have already developed several methods to indirectly estimate ${\text{VD}}_{{{\text{phys}}}} /{\text{VT}}$ without using Vcap (Table 2) [10, 11]. However, the accuracy of these methods is still under debate [11, 42].

### Ventilatory ratio

According to the other form of its equation (Table 2), VR is influenced by ${\dot{\text{V}}\text{CO}}_{2}$ [17–19, 21]. In the early 1990s, authors found that ${\dot{\text{V}}\text{CO}}_{2}$ was a less influential contributor to excess ${\dot{\text{V}}\text{E}}$ compared with dead space in early ARDS [63]; nevertheless, in the era when ECLS is increasingly prevalent, changes in ${\dot{\text{V}}\text{CO}}_{2}$ can be encountered in ECLS-treated patients. Hence, VR is a parameter of great value to patients receiving ECLS; moreover, when making interpatient comparisons, alterations in ${\dot{\text{V}}\text{CO}}_{2}$ caused by ECLS should also be considered. This could account for the results obtained from two recent studies: (a) Morales-Quinteros et al. found that VR cannot be used as an indicator of mortality [25] and (b) Langer et al. employed VR to predict PP responders; however, this attempt failed as well [45].

### End-tidal-to-arterial PCO_2_ ratio

Limitations of this index include the following: (a) The premise of this index is that ${\text{PETCO}}_{2}$ serves as a surrogate for ${\text{PACO}}_{2}$, In ARDS lungs, because the units with different ${\dot{\text{V}}\text{A}}/{\dot{\text{Q}}}$ values empty sequentially, ${\text{PETCO}}_{2}$ is greater than ${\text{PACO}}_{2}$ [16]. (b) This parameter does not take ${\text{VD}}_{{{\text{aw}}}}$ into account. Previous studies have shown that in mechanically ventilated patients, ${\text{VD}}_{{{\text{inst}}}}$ which is an unfixed component of ${\text{VD}}_{{{\text{aw}}}}$ heavily influences ventilatory efficiency [64]. (c) Variations in ${\dot{\text{V}}\text{CO}}_{2}$ and ${\dot{\text{V}}\text{E}}$ receive no attention in this index. For patients treated with ECLS, different extracorporeal blood flow values would induce disparities in ${\dot{\text{V}}\text{CO}}_{2}$. Additionally, ${\dot{\text{V}}\text{E}}$ would rise in proportion to increased ${\dot{\text{V}}\text{CO}}_{2}$.

## Future outlook

### Optimization of the subclassifications of ARDS

Although ${\dot{\text{V}}\text{E}}_{{{\text{CORR}}}}$ failed to identify a subgroup of patients with more dismal outcomes, emerging clinical studies have revealed that a group of dead space ventilation-related indices can provide prognostic information for patients with ARDS [2, 17–22, 24, 25]. Furthermore, adding these indices to the Berlin definition has been demonstrated to improve predictive validity [11, 21]. If their prognostic value could later be confirmed in large-scale randomized controlled trials, dead space ventilation-related indices may be reconsidered when experts update the definition of ARDS to optimize subclassifications in the future.

### Identifications of ARDS subphenotypes to achieve precision medicine

Before the outbreak of COVID-19, to enhance personalized therapy, several approaches for identifying subphenotypes were proposed [65]. After Gattinoni et al. recommended that COVID-19-ARDS be divided into phenotype L (i.e., high Crs) and phenotype H (i.e., low Crs) [7, 46], one study group found that this atypical subphenotype with preserved Crs existed in non-COVID-19-ARDS [66]. Recently, Wendel Garcia et al.identified two subphenotypes characterized by different ${\text{VD}}_{{{\text{alv}}}} /{\text{VT}}$ ratios that responded differently to standardized recruitment maneuvers and had disparate clinical outcomes [67]. Therefore, identifying subphenotypes based on these dead space ventilation-related indices makes it possible for the treatment strategies of ARDS to move from a one-size-fits-all pattern toward a more effective and individualized pattern.

## Conclusion

Over the past decades, since the significance of dead space was emphasized, a large number of innovative dead space ventilation-related indices have emerged. These parameters inform intensivists about ${\dot{\text{V}}\text{A}}/{\dot{\text{Q}}}$ mismatch, thus assuming a pivotal role in PEEP titration, PP andECLS. With the advent of precision medicine, the management of ARDS is rapidly changing, and dead space ventilation-related indices will return to the forefront of research and clinical practice.

## Data Availability

Not applicable.

## Abbreviations

ARDS: Acute respiratory distress syndrome
COVID-19: Coronavirus disease 2019
PEEP: Positive end expiratory pressure
PP: Prone position
ECLS: Extracorporeal life support
${\text{PaO}}_{2} /{\text{FiO}}_{2}$: Arterial partial pressure of ${\text{O}}_{2}$ to fraction of inspired ${\text{O}}_{2}$
${\dot{\text{V}}\text{E}}_{{{\text{CORR}}}}$: The corrected minute ventilation
${\text{VD}}_{{{\text{Bohr}}}} /{\text{VT}}$: Bohr’s dead space fraction
${\text{VD}}_{{{\text{aw}}}} /{\text{VT}}$: Airway dead space fraction
${\text{VD}}_{{{\text{B}} - {\text{E}}}} /{\text{VT}}$: Enghoff’s modification of Bohr’s dead space fraction
${\text{VD}}_{{{\text{phys}}}} /{\text{VT}}$: Physiological dead space fraction
$${\dot{\text{Q}}}_{{{\text{VA}}}} /{\dot{\text{Q}}}_{{\text{T}}}$$: Venous admixture
${\dot{\text{V}}\text{A}}/{\dot{\text{Q}}}$: The ratio of ventilation to perfusion
VR: Ventilatory ratio
$\frac{{{\text{VD}}_{{{\text{alv}}}} }}{{{\text{VT}}_{{{\text{alv}}}} }}$: The ratio of alveolar dead space to alveolar tidal volume
$\frac{{{\text{PETCO}}_{2} }}{{{\text{PaCO}}_{2} }}$: End-tidal-to-arterial PCO_2_ ratio
MIGET: Multiple inert gas elimination technique
ALPE: Automatic lung parameter estimator
EIT: Electrical impedance tomography
${\text{P}}\left( {{\text{a}} - {\text{ET}}} \right){\text{CO}}_{2}$: Arterial minus end-tidal CO_2_ gradient
ECCO2R: Extracorporeal CO_2_ removal
${\text{VD}}_{{{\text{alv}}}} /{\text{VT}}$: Alveolar dead space fraction
vv-ECMO: Venovenous extracorporeal membrane oxygenation
Vcap: Volumetric capnography
$${\dot{\text{V}}\text{CO}}_{2}$$: CO_2_ production
$${\dot{\text{V}}\text{E}}$$: Minute ventilation
Crs: Compliance of respiratory system
